# How experts on Autoinflammatory diseases classify Periodic Fever, Aphthous stomatitis, Pharyngitis and Cervical Adenitis (PFAPA): preliminary results of the Eurofever Delphi survey

**DOI:** 10.1186/1546-0096-13-S1-P145

**Published:** 2015-09-28

**Authors:** F Vanoni, S Federici, S Ozen, J Frenkel, H Lachmann, A Martini, N Ruperto, M Gattorno, M Hofer

**Affiliations:** 1 Istituto G. Gaslini, UO Pediatria II - Reumatologia, Genova, Italy; 2 CHUV, University of Lausanne, Pediatric Rheumatology Unit of Western Switzerland, Lausanne, Switzerland; 3 Hacettepe University Faculty of Medicine, Department of Pediatrics, Division of Rheumatology, Ankara, UK; 4 University Medical Center Utrecht, Department of Paediatrics, Utrecht, Netherlands; 5 National Amyloidosis Centre, London, UK

## Introduction

The diagnosis of PFAPA is currently based on set of criteria proposed in 1999 and known as the modified Marshall's criteria. Pediatricians rely on their own clinical experience to diagnose PFAPA, but no validated set of classification criteria has been established up to now.

## Objectives

To understand how physicians involved in the clinical care of patients with Autoinflammatory diseases (AIDs) classify patients with PFAPA in daily practice.

## Methods

by using the Delphi and Nominal Group Technique, we started an initial phase of three following e-mail surveys. In the first survey, clinicians/biologists or other health professionals working in the field of autoinflammation were asked to identify the variables that they consider as important, in the current clinical practice, for the diagnosis of patients with PFAPA. This survey was open to not influence the experts.

## Results

We sent the first survey to 124 experts. The overall rate of response was 107 (86%): 101 experts responded to be interested in the survey, 76 completed and confirmed it, 6 responded not to be interested. There was any clinical variable chosen by all the participants. The five most cited clinical variables were periodic fever (80% of experts), cervical adenitis (79% of experts), aphtous stomatitis (76% of experts), pharingotonsillitis (71% of experts), abdominal pain (24% of experts).

The increase of acute phase reactants during episodes and the response to steroids were proposed as interesting variable by 44% and 43% of experts respectively. The exclusion of the inherited genetic periodic fever by genetic test is important for a little part of experts (24%). Response to tonsillectomy was cited by only one expert.

## Conclusions

The preliminary results of the first Eurofever Delphi Survey show a high rate of response, underlying the interest of the scientific community in this topic. At the end of the Delphi Survey rounds, we will obtain different set of clinical criteria and we will verify their performance in comparison to already existing criteria in a cohort of patients with PFAPA enrolled in the Eurofever Registry. The final step will be a Consensus among experts (geneticists and clinicians) in order to define the best combination of clinical and genetic data for the definitive classification of patients with PFAPA.

